# “I tried so many diets, now I want to do it differently”—A single case study on coaching for weight loss

**DOI:** 10.3402/qhw.v10.26925

**Published:** 2015-08-14

**Authors:** Reinhard Stelter

**Affiliations:** Department of Nutrition, Exercise and Sports, University of Copenhagen, Copenhagen N, Denmark

**Keywords:** Health coaching, life style change, meaning making, case study, overweight, obesity, diet, physical activity, weight loss

## Abstract

In this single case study, the author presented an in-depth description and analysis of a coaching intervention with focus on weight loss, conducted over 10 sessions in the course of 17 months. The client was a well-educated woman in her late 30s, who had tried many different forms of dieting over the years—with little and no lasting effect. In his coaching approach, the author went beyond a pure behavioural change model, that is, based on the Health Belief Model, and tried to take a whole-life perspective, where the client learned to link specific events and habits in her work life and everyday life with specific eating habits. In their collaborative practice, coach and coachee initiated changes both in regard to diet, physical activity, and healthy life style, in general. In a theoretical section, the change in understanding with regard to overeating was presented. Finally, an intra-active model—viewing the client as a self-reflective individual—was used as theoretical basis. A narrative analysis of the first session and a cross-session examination was presented to show, analyse, and understand the procedure of the coaching approach. Finally, the voice of the coachee was heard in regard to her personal experiences during the process. The data material was based on audio recordings of selected sessions, notes written by the coach from every session, and final written reflections by the coachee.

By now, we all know that overweight, defined by a body mass index (BMI—the weight in kilograms divided by the square of the height in meters) above 25, and especially obesity, defined by a BMI of 30 or more, is a threat to people's health. On the basis of a large cohort study, Adams et al. (2006) could conclude: Excess body weight during midlife, including overweight, is associated with an increased risk of death. The number of people who are overweight and obese is growing in many countries, led by the following top 10: (1) USA, (2) China, (3) India, (4) Russia, (5) Brazil, (6) Mexico, (7) Egypt, (8) Germany, (9) Pakistan, and (10) Indonesia. Worldwide, the number of overweight and obese people increased from 857 million in 1980 to 2.1 billion in 2013—an increase of more than 145% (www.health.usnews.com/health-news/health-wellness/articles/2014/05/28/america-tops-list-of-10-most-obese-countries; retrieved 7 August 2014). The reasons for this development are manifold: lack of energy balance, inactive lifestyle, environmental factors, lack of sleep, just to mention some of the central causes (www.nhlbi.nih.gov/health/health-topics/topics/obe/causes.html; retrieved 7 August 2014). The relationship between socio-economic status and obesity must be nuanced depending on the level of development of the country. McLaren (2007) documented the following as the overall pattern of results in regard to this relationship: For both men and women, an increasing proportion of positive associations and a decreasing proportion of negative associations were found, as one moved from countries with high levels of socio-economic development to countries with medium and low levels of development.

On the basis of these epidemic dimensions, the issue of overweight and obesity cannot be taken lightly. It is probably one of the most fundamental threats that governmental health authorities are forced to deal with. Compared to the size of the challenge, the topic and objective of this article seem to be a drop in the ocean. The attempt here was to present a concrete case study with two central objectives: First, to document and evaluate the client's experiences of the change process, based on the intentions and dialogical strategies of the coach, and second, to document and analyse the process and impact of coaching for weight loss coaching dialogues. The approach presented here used a broader perspective than a sole focus on perception of threat as the basis for behavioural change (e.g., the Health Belief Model [HBM]), because of the author's firm belief that the change in behaviour is based on new ways of sense- or meaning making, of understanding oneself, and of attitudes towards life. This shift in the mindset will slowly prepare the client and make her ready to initiate the first step towards a change of lifestyle.

## Approaches and procedures for behaviour change

During the twentieth century, the understanding of the individual has changed. On the basis of a better understanding of psychological reasons for obesity (Swencionis & Rendell, 2012), this change of understanding also had an influence on the approaches to helping people with weight loss and lifestyle change (Ogden, 2002). (1) During the first half of the century, the main approach assumed that the individual was *passive*, and behaviour could be predicted and influenced through specific stimuli (Pavlov, 1927; Skinner, 1953). (2) From the 1960 onwards, the behavioural law of conditioning was substituted by emphasizing “an interactive alignment of the individual and their environment” (Ogden, 2002, p. 21). Individuals were understood as being able to process information, and on the basis of their cognitive understanding they could make changes in their behaviour. This understanding can be seen in the HBM (Daddario, 2007; Rosenstock, 1974). Very briefly described, the model stated the following: Perceived seriousness and perceived susceptibility with regard to specific health threats might lead to growing awareness of possible health threats, which might enhance the likelihood of engaging in health-promoting behaviour. In a further development of the model, self-efficacy (Bandura, 1977, 1997)—the confidence that a desired behaviour can be carried out—was included as an important factor for change (Becker & Rodenstock, 1987; Rosenstock, Strecher, & Becker, 1988). (3) In a way, the further development of the HBM was the first indication of the third phase in the understanding of the individual; a phase that was inspired by poststructualism and which had become more and more prominent during the last two decades. Ogden (2002) described the shift by presenting *the reflexive, intra-active individual*, a term fairly new in the literature, which Ogden (1995) had already described as follows:

The contemporary intra-active individual is characterized by an agency and an intentionality which is directed internally towards their inner self. The late twentieth century object of psychological thought has become a subjective entity whose subject is the self. (p. 412)

The neologism “intra-active enactment,” introduced by Barad (2007), who was inspired by Michael Foucault and Niels Bohr, was related to the idea of a growing subjectification in today's society. Højgaard and Søndergaard (2011) wrote:

The concept of subjectification builds on the work of Michel Foucault, his conceptualisation of discursive power as productive, and his point about subjective submission under discursive power as a process which involves a simultaneous production of subjective existence and agency. The humanist idea of a “core self” is radically transgressed in this line of thought and replaced by the idea of an emerging subject. The basic figure of simultaneity between submission and agency in the formation of the subject has had an enormous impact on poststructuralist thinking over the past decades. (p. 340)

In that sense, subjectification is a concept that describes individuals as agents who simultaneously shape whatever they are shaped by. In a similar way, subjectification describes the growing impact on the individual's handling of specific social and personal challenges in regard to health issues such as smoking, diet, weight loss, and involvement in physical activities and sports. In regard to these challenges, the subject tends towards various forms of *surveillance*, a phenomenon that Foucault described as constant and pervasive forms of observation that finally lead to growing individual self-control. Individuals become their own “worst enemy.” From this perspective, there are indications in the literature of the critical influence of monitoring weight on psychological states (Dionne & Yeudall, 2005; Ogden & Whyman, 1997).

## The intra-active overeater

**Figure 1:**
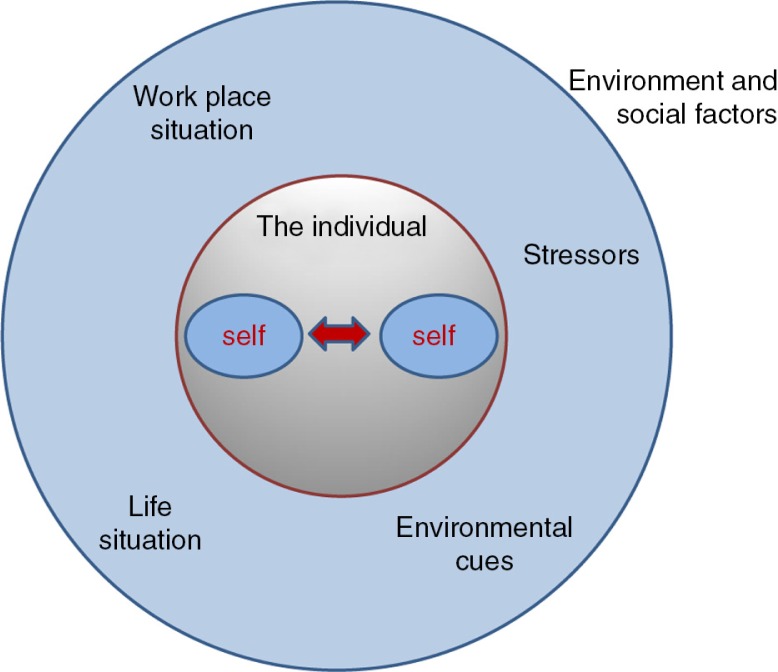
The intra-active individual.

On the basis of this intra-active model of the self, Ogden (2002) described the change in regard to the understanding of dieting as follows. In the 1960s and 1970s, the psychological understanding of overeating was influenced by the idea that overweight and obesity were highly and sometimes uncontrollably responsive to external and environmental cues such as time of day, sight, number and salience of food cues, and taste (Schachter, 1968; Schachter & Gross, 1968). In the last quarter of the twentieth century, new models emerged. Ogden (2002, p. 87) referred to studies conducted with normal weight individuals, studies which “suggested that trying to eat less (i.e., restrained eating) was a better predictor of food intake than weight *per se*.” A theory of restrained eating behaviour evolved. *The perception of weight*, a biological construct, was no longer seen as the main determinant of eating behaviour. Instead *restrained eating*, a psychological construct, was introduced as means to evaluate food intake (Herman & Mack, 1975; Hibscher & Herman, 1977). In consequence of this approach, overeating could be characterized as a *failure of self-control* (Ogden, 1997, 2002); the individual does not manage to keep him- or herself under “proper” surveillance. This led to the development of to an *escape theory* to explain disinhibition with the consequence of overeating (Heatherton & Baumeister, 1991): Binge and overeating arise as part of a motivated attempt to escape from self-awareness and self-control in situations where one's own high standards and demanding ideals are put under pressure. Eating turns into pure relief for surveillance and becomes self-satisfactory. In a study of Heatherton and Baumeister (1991, p. 101) binge eating was also “associated with decreased negative affect.” On the basis of this understanding, Ogden (2002, p. 89) concluded: “Therefore, a shift to lower self-awareness resulted in reduced self-control”—with the final possible consequence: overeating. Her definition of the individual “as a reflexive, intra-active and self-controlling self, who is not biomedical and not social” (Ogden, 2002, p. 97) was an attempt to overcome the dualism or disintegration between social and environment dimensions on the one side, and individual and psychological dimensions on the other side. With this model, the intention was to merge the individual with social and environmental factors. Individuals become reflexive and intra-active subjects and objects of their own self-reflection (Figure 1). Indubitably, environmental factors have an impact on the individual but not in a simple causal relationship.

Højgaard and Søndergaard (2011, p. 347) saw the concept of intra-action as “premised on a conception of radical co-constitution of subjects as part of material-discursive enactments.” Intra-active theorists see individuals embedded in a specific social discourse of, for example, being slim, looking good, controlling one's weight, being physically active, etc. as well as in discourses such as “when I am unhappy or under pressure, eating helps me to feel satisfied.” All these discourses generate intra-active movements in the individual as a form of material-discursive practice, for example: “I eat fast food or sweets to calm down when I come home after a stressful day at work.” This practice becomes meaningful for the individual. Repetition underlines this meaningfulness and becomes the motor for the performativity of, for example, binge eating. The concept of intra-action takes the complexity of the individual's world into account by embracing the diverse factors of the material, social, and subject world.

This theoretical understanding was assumed to be helpful for the further understanding of coaching as a dialogical tool for self-reflection around eating and striving for weight loss. Coaching should be seen as an integrative part of the individual's intra-active process where new material-discursive practices are initiated; practices that simultaneously express and enact new meaning and understanding in regard to the complexity of the client's life. The intention of coaching for weight loss should replace self-surveillance with a self-understanding that helps clients manage their own life in a way that enables new dimensions in regard to handling specific challenges.

## Coaching as the basis for changing eating habits

If we see the lack of self-control as the greatest hindrance to weight loss, then individuals who wish to change their eating habits have to get involved in a *self-reflective process*, in our case offered through coaching, which may be seen as the basis for a new material-discursive practice. Based on the presented intra-active approach, it would *not* work simply to focus on the goal of weight loss and pursue the direct path towards a change of behaviour. A goal focus on weight would only keep the person's attention on self-surveillance and self-control. It is not enough to understand the perceived seriousness and the perceived susceptibility to personal health issues, for example, in regard to overweight (as suggested by the HBM). Instead, individuals striving for weight loss are invited by the coach to see themselves from a new perspective and somehow learn to grasp the complexity of their world in a new way. They have to take a closer look at themselves as being co-constituted as both a subject and object of their lived practice.

In the following, a definition of health coaching is presented on the basis of the current review of the literature. Further on, the author will unfold the fundamentals for coaching for weight loss that takes the *approach of the intra-active overeater* into account, and that is seen as the more effective approach than the traditional narrowly goal-oriented approaches.

Wolever et al. (2013) have published a systematic review of the literature on health and wellness coaching, observing an emerging consensus in what is referred to as health and wellness coaching. On the basis of their literature study, they define health and wellness coaching as

a patient-centered approach wherein patients at least partially determine their goals, use self-discovery or active learning processes together with content education to work toward their goals, and self-monitor behaviors to increase accountability, all within the context of an interpersonal relationship with a coach. (p. 52)

They continue by presenting patients or clients as “as lifelong learners whose individual personal values and innate internal resources can be cultivated in the context of a supportive relationship to guide them toward their own desired vision of health” (see also Smith et al., 2013, p. 52). Based on this understanding, an approach to coaching for weight loss will be unfolded by emphasizing the *self-reflective* dimension of the dialogue, by highlighting *personal values and meaning making* and finally, as a result of this reflective process, by preparing for change of behaviour (see also Stelter, 2007, 2014). The use of this approach will be illustrated in the later description and analysis of the case study with Anna. In the following, a number of central criteria for this self-reflective coaching for weight loss approach will be presented:

1. *Raising the level of the dialogue to meaning making*: Making meaning of life events, action patterns, and specific habits—good or bad—is the pivotal point of a self-reflective coaching dialogue. Meaning emerges twofold by interlinking individual with social meaning making. Based on phenomenological thinking, individual meaning making unfolds by making sense of specific experiences and (bodily) sensations, by bringing the pre-reflective (e.g., habits and routines) to the surface of the reflections in the dialogue (Stelter, 2000, 2008; Stelter & Law 2010). Learning to be mindful by sensing what happens in specific situations and in regard to (re)establishing new habits is a central dimension of individual meaning making.
  Based on social constructionist thinking, social meaning making evolves by making sense of and reflecting on the impact of specific social relations, specific others, and the social context in general on one's identity and one's way of being in the world (Gergen, 2009). We co-construct the world through and in the relationships with others.
2. *Focusing on values*: Values embrace the most central and fundamental issues in our life and are—often implicitly—guiding markers for our way of acting in the world. Too often, coaching takes its point of departure in a specific goal [e.g., in the GROW model (Goal-Reality-Options-Wrap-up; Whitmore, 2009), or in motivational interviewing], but goals are at the lowest level in the hierarchy of intentionality (Stelter, 2009, 2014). On the next higher level, “purpose” is the dimension that goals are influenced by, and on the crown floor of the hierarchy of intentionality we find the dimension of meaning making, which inevitably leads to a reflection on values (Stelter, 2007, 2014). To do successful coaching interventions, it is important to build bridges between the clients’ ambitions to change their way of living and their specific individual values, which they can investigate on their journey, and which in the end can lead to a change of behaviour.
3. *The narrative-collaborative dimension of coaching*: Meaning and values are often embedded in specific episodes and events. As part of a transformative learning process (Illeris, 2004; Mezirow & Associates, 1990), where learning always implies an impact on identity and self-understanding, coaching becomes a collaborative enterprise between coach and coachee. The narrative element is highlighted by connecting the coachee's actions with identity issues and vice versa. Narratives are the vehicles which link specific events in a timeline and which have a special impact on the client. If these narratives are a strain on the client, the aim is to *deconstruct* them in the collaborative process between coach and client. Deconstruction implies the potential for change. By reflecting on the narrative and presenting additional possible interpretations, the dialogical partner applies procedures that undermine the taken-for-granted understanding of the client's life and identity (White, 2004).

The author was aware of the close connection between the self-reflective dimension of the dialogue and the focus on the final goal: weight loss. The path in the GROW model, or motivational interviewing (Rubak, Sandboek, Lauritzen, & Christensen, 2005) appeared to be too goal-focused with too little attention paid to possible barriers and the complexity of life, which are more thoroughly dealt with in an in-depth, self-reflective coaching process based on the three key areas presented above.

## Methodological reflections in regard to the single case study

In this case study, the intention is to present the whole picture of a single case on coaching for weight loss (more on case study design, Yin, 2009). The research documentation in the form of this article was NOT at all planned beforehand; it was only decided a few weeks before the last session in the coaching process.^1^ As mentioned earlier, the objective of this case study is twofold: First, to document and analyse the process and impact of coaching for weight loss dialogues, and second, to document and analyse the client's experiences of the change process, based on the intentions and dialogical strategies of the coach. To be able to meet these objectives, the following data material was included:

1. Notes taken by the coach during each session (this is a standard procedure).
2. Audio files of the first six sessions (it was actually the client who asked for permission to record the sessions).
3. Reflective notes, produced by the client before the final session.

The material is used in different ways. To provide a basic understanding of the coaching process, the first session is presented as a narrative that is developed on the basis of an analysis of the coach's notes and an audio-recording of the session. The rest of the process is examined in a critical analysis of the coach's notes and the audio-recordings. Finally, the client's reflections are presented in their original form as a written narrative submitted to the author. In the concluding section, the author's and the client's respective analyses are compared, and some final conclusions are presented.

### Narrative analysis of the first session

The narrative presentation of the first session unfolds on the basis of an analysis of the researcher's notes and of the audio-recording of the session. Specific events and situations were chosen and highlighted to make the narrative coherent while remaining faithful to the data. This process of shaping the storyline was part of the interpretation and analysis of the qualitative material. As in any qualitative analysis, a major effort is done to achieve credibility. The intention was to use Richardson's (2000, p. 923) idea of writing and storytelling as a way of analysing interview material: “Writing is also a way of knowing—a method of discovery and analysis. By writing in different ways, we discover new aspects of our topic and our relationship to it. Form and content are inseparable.” The narrative–analytical perspective used was that the researcher had to develop meaning out of the material.

### Cross-session analysis

Subsequently, the entire sequence of sessions was analysed by coding the notes and the speech from the audio-recording of the session, thus extracting the central meaning and forming central themes that represent the core issues of the series of coaching sessions. The idea of writing the narrative down was also applied in this section by combining the presentation of themes with a reflective analysis that includes the theories presented earlier.

### The case study as practitioner research

The case study should also be understood as the work of a practitioner researcher (Jarvis, 1999) or a scientist practitioner (Lane & Corrie, 2006), meaning that the author sees himself both as a researcher and reflective practitioner (Schön, 1983; in regard to coaching: see especially Stelter, 2014). Lane and Corrie (2006) defined the *ability to formulate the actual case* based on its inherent challenges as one of the most essential qualifications of a psychologist or a coach. They saw this process as a form of *psychological sense-making*, which can be understood as a central criterion for reflective practice and thereby for the development of expertise as professional.

## Case study: Anna's path towards weight loss

In this single case study, the author presents an in-depth description based on an analysis of a coaching intervention with focus on weight loss, conducted over 10 sessions spanning the course of 17 months. The last session was in September 2014. The client, we call Anna, a well-educated woman in her late thirties, has tried many different forms of dieting during her adult years—with little and no lasting effect. First in the very final phase of the coaching process, the author decided to publish this work—with the full approval of Anna, who even agreed to participate in this publication with her own reflections written just before the last coaching session, a reflection that was also included in the session to help her planning her future path.

Anna contacted me after she had participated in a coaching workshop of mine, where she became very interested in my work and my approach. Through email, we agreed on having at least five meetings, but we ended up having 10. Her decision to start coaching for weight loss with me was supported by her conviction that my coaching approach might help her more than all her former attempts and strategies towards weight loss, which had not really satisfied her, and which did not live up to her expectations in regard to losing weight and maintaining the success. She also stated a fundamental principal for her: She does not want to lead her life in a state of permanent dieting. Food intake should be a pleasurable part of her life. Her ambition was to lose up to 25 kg. Here is the narrative of the first session, a narrative that shall also help the reader to get a basic impression about how coaching was conducted:

## The first session

A Friday afternoon in April 2013, Anna enters my office. She quickly starts talking about her life. She feels that since her childhood she has eaten too much candy, especially when spending time with her grandparents, which she has done quite a lot. When her grandmother died, she was quite depressed for a long period of time. “But I ‘woke up’ again and actually lost weight. I overcame it, and life was more ‘bubbly’ and good. But after writing my master's thesis I have gained weight again. I lost control. I must get it stopped! But I have a hard time doing it myself,” she states with a hint of despair.

She begins to talk about her work: She says that she should actually be very pleased with her job. She uses her university degree and has a good position in the organization. “But I feel sapped. I don't use myself in the right way. I am not on the right shelf. My job holds no meaning for me.” In spite of this slightly discouraging assessment, she at the same time ensures me that her boss is very pleased with her. She considers herself to be a very valuable and well-liked employee. “Even though I am very much a perfectionist when it comes to my work and often work both in the evenings and the weekends, I don't experience my job as being meaningful! I just can't do my best.”

She tells me that she has gone through a short coach training in 2010 and would like to develop a concept concerning coaching the 60 plus age group, where she would like to help people in their transition from work life to retirement, “the third age.” She feels that she has developed many competencies within the field of coaching, and she does work with coaching on a small scale. “I began to see the world in a different way,” she says. “But the coach training has made me feel that it's wrong for me to be doing my current job.”

We talk about what it is about coaching that attracts her, and how coaching helps her experience meaning. She tells me that she has always been interested in performance art, relations, and other people. It is within this field, she wants her future to be.

She talks about the challenges she faces in regard to her weight. “I strive for the perfect image. I either lose weight for real or else I might as well not bother!”—“But I have the feeling that I don't take myself seriously.”

I ask her to tell me more about her job. She says that she often works up to 60 h a week (and here we are talking about a regular “37 h a week” job more or less.). She explains that she often works more than her boss, who usually goes home around 4 p.m. and doesn't read emails during the weekend. In a humorous way—with the hope she will be slightly provoked in a good way—I say to her: “So sometimes, it is as if you have IDIOT stamped on your forehead when you see your boss go home before you.” “Yes, actually, you are right; I can see that sometimes I do have IDIOT stamped on my forehead.”

“To me it seems like you don't take proper care of yourself,” I say with a tone of understanding. “Yes, those words are very fitting: I need to take proper care of myself,” Anna confirms my statement, clearly impressed by how well I capture the essence of her life situation. I talk a little more about taking care of one's self: “It is also manifested in the way you take care of yourself, also in regard to cooking, exercise, and a healthy lifestyle. It is important that you enjoy things and allow yourself the time for it.” “Yes, you are absolutely right, I don't take myself seriously.” To get a sense about taking herself seriously, I ask Anna the following question: “On a scale from 0 to 10, where 0 is ‘I don't take myself seriously’ and 10 is ‘I take myself very seriously’, where are you in regard to work on the one hand, and in private on the other?” Anna answers with conviction as follows: “At work it is a 9–10 and in private 3–4.”

We end up agreeing on the following assumption: The attitude I have towards eating is a reflection of the attitude I have towards myself.

I ask about her eating habits on an ordinary day, which she describes as follows:

- Breakfast: two pieces of crisp bread and a glass of milk
- Lunch: salad and a little bit of fish
- When I get home around 6:30, I don't have energy for much else than a pizza or a pita with chicken and a large Pepsi Max. The “healthy version” is that I eat rye bread. And a bag of candy with a movie is her idea of peace, happiness, and relaxation.

We are getting close to the end of the session, and I am very interested in getting concrete about suggestions for behavioural change: “How about if you substituted your Pepsi Max with water?”—“Ah, then I would like it to be sparkling water because of the fizz,” Anna answers in a supportive and motivated manner. “But could it be okay with Pepsi Max on the weekends?” she asks. I try to describe, how this hope of getting Pepsi on the weekends impairs her ability to fully enjoy the taste of sparkling water—“So you can make it to the point where you almost don't even like Pepsi anymore.” “Actually, Pepsi sometimes has a weird taste of iron—so you're right actually. I don't always like it!”

Anna suddenly seems very motivated to establish MORE different changes in her life. We agree on the following list:

- Only sparkling water instead of Pepsi
- Dark chocolate instead of candy
- Go home earlier from work
- Go for a walk—in the time where I used to stay late at work

Finally, I ask her about how she has experienced the session and our collaboration. “The most important thing for me was the part about taking care of myself. I really liked when you said it says IDIOT on my forehead. I need you to push me a little bit,” she states. I am almost a little surprised that she appreciates my slightly provoking comment in such a prominent way. I had not expected that.

We agree that we will arrange the next meeting via email, because Anna does not have her calendar with her. For this first meeting, she actually came on her day off.

## Reflection on the session

In this section, I would like to see myself as a practitioner researcher/scientist practitioner, and thus I would like to reflect upon some of my main theoretically based positions and strategies, and I also would like to include some statements from Anna's feedback that I received during the very last session:

In the beginning, I tried to get a picture of Anna's life and biography, especially is the parts which were relevant for her ambition to lose weight. The close interconnectedness between specific life events and eating habits became obvious. As the coach I became the intra-active partner by helping her to understand herself in a new way. We focused on specific values, on what was important for her, and how she sometimes struggled with being her. In this collaborative dialogue the intake of sweets and candy could be connected to a state of mind where she felt *safe, secure, and trusting*. Sweets and food in general had certain significance in her life, which in the end resulted in an unhealthy, uncontrollable, and unreflected food intake. As mentioned earlier, overeating can be “associated with decreased negative affect” (Heatherton & Baumeister, 1991, p. 101) and with avoiding negative states of mind—the *escape theory* that explains the avoidance of self-awareness in situations where one's own high standards and demanding ideals are under pressure.

Anna's life situation and material-discursive practices around food intake were strongly influenced by her working life situation. During our coaching conversation we reached the conclusion that a long working day had a negative influence on the way Anna would “take care of herself”—a fact that also had an impact on her food intake. In our final session, Anna concluded that regular working hours without overtime help her very much to keep up a healthy lifestyle and appropriate eating habits. In that sense, Anna's decision of working less was a helpful strategy for her to prevent overeating that arose as part of a motivated attempt to escape from self-awareness in situations where her own high standards and demanding ideals were put under pressure (see the earlier exposition of the escape theory).

In the last phase of the session, our dialogue changed from investigative and reflective with a focus on developmental issues, towards concrete collaboratively developed guidelines that aimed to help Anna reduce her food intake in a way which corresponded with her wish that lifestyle change should not to be associated with a permanent state of suffering. My intention was to negotiate suggestions which Anna could accept as substitutes for high-calorie products (e.g., mineral water instead of cola). Anna and I put focus on collaboratively developed recommendations as a part of most of the following sessions. What we came forward to was a number of guidelines that were not based on a position of pure self-control, but these guidelines were the result of a longer reflective process, where Anna became clear about how her practice in regard to food intake was influenced by especially her work situation. In that sense, the guidelines were the result of a process where they appeared as meaningful to Anna, and where they were much more than just as a diet instruction. In our very last session, Anna evaluated that it was very important to her to realize that not everything was negotiable. From the beginning, she felt that I was rather determined in regard to substituting cola with mineral water. But my invitation to self-reflection and her growing self-understanding were the most important aspects of our coaching which made her ready for change.

From the theoretical perspective, the session had two central phases: (1) in the first and longer part, the main focus was to give Anna new opportunities for being an intra-active individual; thus, Anna became aware of the way her life situation (e.g., long working hours etc.) had shaped her and, ultimately, her habits and (2) specific material-discursive practices (e.g., buying food, non-cooking, food intake, reading while eating) had shaped her in a specific way and put her on a path towards overeating, which eventually resulted in overweight.

## Milestones of the further process—a reflective analysis

In the following, I present some key themes that emerged from an analysis of the collected material (notes by the coach, audio recordings) and which could thus be identified as key issues and milestones that helped Anna understand herself differently and progress in regard to lifestyle changes. The presentation of these milestones is combined with theoretical reflections alongside reflections informed by the intra-active model.

### Searching for a new job

In the second session, 1 month after the first, Anna told me that she had applied for two jobs and has been invited for interviews at both places. She experienced this situation as a huge dilemma, especially in regard to having to say no to possibly one of the two. “I fell back and drank cola, just to please myself and to care for myself. I am quite afraid to disappoint people, and drinking cola is a way for me to cope with the situation,” is how she described her feelings in the beginning of the session. I got the impression that she was already quite aware about specific mechanisms in regard to her unhealthy eating habits, and we had a longer conversation about how she could *take care of herself* in other ways.

She also told me with pride that she had actually renounced one of the two job offers, because in the end she did not like the job, and she was happy to accept the offer from the other employer. While remembering the feelings in regard to her work situation from the first session, I congratulated her on her great initiative and the big success of taking responsibility of her own life. “Now I just have to stick to my goal that I do not want to work overtime. I have talked about this in the interview, and they accepted me on these terms anyway. So, now I can only hope that they keep this in mind when the situation comes,” were her final thoughts about this issue.

Concluding this topic, the following can be stated: Anna had already taken a big step forward by changing her job situation and by being serious about specific issues, for example how working overtime in her current job had had a negative impact on her lifestyle. From the perspective of the intra-active model, Anna had a hard time in the first phase of the process in regard to living up to her high personal standards (e.g., not disappointing others). Food intake became a way for her to calm down and achieve a sense of safety. Coaching helped her to abandon some of her old intra-active dialogues: She became aware of how drinking cola was a surrogate for *taking care of oneself*.

### Change of attitude towards work

During several sessions, her work situation was the focus. Already in the first session, Anna became aware that her perfectionism, or seen from another angle: her anxiety of not being good enough, of not living up to her ideals, of losing control, were central factors in regard to keeping bad eating habits alive. On many occasions, the pressure she felt at work led to forms of binge or over eating, where she used food to calm herself down and first afterwards noticed what had happened to her. As Heatherton and Baumeister (1991, p. 10) argued, eating was “associated with decreased negative affect.” During several sessions, we reflected on how Anna could handle job pressure in a different way. She came to the conclusion that working overtime had to be banned, and we also reflected a lot about how she could learn to accept herself as a *good enough worker* by appreciating her own work effort and by fighting bad conscience (5th session). I introduced her to writing a “scrapbook” based on the following task: Write down three things in the evening which you succeed with or were happy about during the day. Through coaching I invited Anna to develop a “new” intra-active individual by helping her to understand herself in a new way, thus supporting her in developing new material-discursive practices that could help her to view and ultimately handle specific work situations in a new way. Furthermore, by introducing a “scrapbook,” I helped her appreciate herself more and become aware that she actually was not far from living up to her ideals.

### Being mindful

We worked quite a bit on being mindful. During the first session, Anna told me about her habits. Preparing food had no high status in her life. Often, when coming home from work late, she just bought a pizza or other types of fast food from a corner shop, plus a big bottle of cola, and while eating she used to watch TV or do further work or reading on her computer. Even when she did her own cooking, she did not seriously value her own effort. Also here, she combined eating with reading or watching TV. I made an “irreverent”^2^ statement: “So, the dining Anna does not give much respect to the cooking Anna!” We talked about possible consequences (e.g., the amount of food she might eat), it could have been that she was not thinking about or, to put it even better, fully sensing her dining. I introduced her to the mindfulness exercise where you eat a raisin very slowly and mindfully (www.staroversky.com/blog/mindfulness-exercise-eating-a-raisin-mindfully, retrieved 20 November 2014). The exercise was an eye-opener.

We developed an agenda for good habits: No TV while eating, be attentive when eating, enjoy the nuances of your food, be in the here and now of the situation, enjoy the pleasure of your own company—and slowly Anna changed her attitude and habits on the basis of new and positive experiences. She felt that she was taking herself much more seriously and taking better care of herself.

Inspired by the intra-active model, developing these new habits became the basis for shaping a new understanding about her material-discursive practices around eating. These new practices were also the basis for understanding and shaping herself in a new way—somehow becoming a *different person*, because the meaning associated with eating appeared to unfold in a new way.

### Making plans—establishing alternatives

As in the end of the first session, we frequently made plans that helped Anna focus on specific aspects of lifestyle change, for example, Monday to Friday: no warm food in the evening, only one to two slices of bread with salad and one piece of fruit, walk to and from work twice a week, practice the raisin exercise every evening, and take a long walk during the weekend. She decided to work on a painting on the topic “my wishful state, my best possible future,” which we talked about in the following session.

These plans were like a contract between us. They helped keep her on track. We celebrated her successes. And if there were objectives she struggled with, we reflected about her challenges.

We developed alternatives together, for example, breathing like in yoga, especially when she experienced a situation as stressful; baking and eating stone-age bread (only with nuts, sesame, etc.—no flour); and inviting her sister to be a buddy to help keep her on track. After 4 months, Anna started to jog or walk every day. At this time, she also decided to begin with the 5:2 diet (Mosley & Spencer, 2013), which her sister (with no weight problems) already practiced.

Here, the focus was on her material-discursive practices, which also shape the individual's development. By changing these practices, the individual gets a chance to act and, somehow, to be different. A change of practices in regard to how to eat and live one's life has an impact on the way a person is able to see herself. As our sessions progressed, Anna felt increasingly ready for the changes that were necessary for her to succeed in “reaching her goals,” which basically revolved around developing new practices that were established as meaningful in regard to her life and the life she wished to construct. Gradually, we developed a working relationship, where I as the coach became a *fellow human being* (Stelter, 2014) and an active supporter of the change process.

### It is not a cure—it is my life!

This sentence was presented by Anna during the last session, where we were looking back and evaluating the course of our working relationship. Changes are not just a cognitive decision; they have to make sense in the actual world of the client. Changes have to grow based on the reflective process about the lived challenges of the participant. Anna has learned that her binge or over eating was actually closely related to insecurity, loss of control, lack of self-confidence, or stressful life events. During one of our later sessions, she remembered a situation when she was little and her father—who was divorced from her mother—asked her to cook for both of them. Although her father did not have bad intentions, Anna felt terribly pressured by this responsibility. She wanted to do her best but felt that she could hardly handle the pressure. Remembering (Stelter, 2014; White, 2007) this early event and articulating her feelings suddenly made her ready to understand a lot of later experiences in her life; events that she had reflected on since the first of our sessions. This story really made her ready to see herself in a new light. On the basis of whole-life perspective, weight loss is not just a matter of diet, physical activity, and eating habits. Bad habits have their story, their good reason. When Anna learned to understand her story, the path of development and change was made possible.

In conclusion, this episode and the whole course of sessions are to be examined in light of the intra-active model that was presented as the theoretical framework. Generating meaning in a new way and through the collaborative process between Anna and the coach was regenerated in and through the intra-active dialogue with Anna who was able to reflect on herself and her practices in a new way. In collaboration with the coach, Anna was a narrator of specific events and life situations. In this course of coaching session, the intra-active dialogue was supported by a collaborative partner, the coach. Meaning was shaped through reflecting, re-telling, and remembering of different life situations and specific practices that seemed understandable but inappropriate. This process of achieving a new understanding of oneself and of having a partner in one's intra-active dialogue can be seen as the very foundation of change. The goals that were formulated in specific situations needed to be anchored in a new understanding of what was seen as meaningful and, at the same time, anchored in specific values that were important to the individual and which could be translated into action through specific material-discursive practices.

## Anna's final reflection on the coaching process

When I, as the author, decided—with informed consent of Anna—to write this article, I also asked Anna to write an honest final reflection about her perspective to our course of coaching sessions and the impact they had on her. This is what I received from her.

### Goal and results

After around a year and a half of coaching, I have become 12 kg lighter. It is a great relief and that's why I choose to use the term “becoming lighter” instead of “losing weight.”

My original goal when I first started was—as far as I remember—to lose 20 kg or a little more than that. I haven't reached that goal yet, but my life has changed in so many other ways, it feels like the goal has been reached. The goal has changed during the coaching process. It quickly became clear to me that it wasn't going to be a diet where I, after my goal was reached, put all the weight on again and maybe even more. That has happened before. This time it was going to be a lasting change in lifestyle.

That point has been quite central in the process, as the goal is more about having a normal relationship with food than losing weight. But that also means that the weight loss hasn't gone as quickly as if I had been on a diet. A challenge in the process has been—and will most certainly continue to be—finding the balance between cutting back on junk food and sweets and also finding a level that is possible to adhere to for the rest of my life. An important point that became clear during the coaching was that it is not about losing something or restricting myself from the things I like. It is more about the amount and trying to come up with good and delicious alternatives, so I don't feel like life is tough and unfair, because I always have to say no to the things I want. The coach made it clear to me that it is all about doing something good for myself.

### Added bonuses

Other goals I have reached in the process are: becoming healthier, also in the long run, more mindfulness in my daily life, not only when I eat. One of my underlying goals was to start exercising again—running and playing tennis. I have started running again and besides, I have found joy in walking to and from work—a couple of hours every day.

The most important thing has been the feeling of coming out of a deep dark hole. I have gone from having an inner voice telling me every day that I must eat sweets or the like, to feeling like I have a choice—I can choose to eat it or I can choose not to and do it another day. That is the greatest relief.

### Process and theme

How has this happened then? Conversations with the coach about habits and automatisms, presence and being mindful, my childhood, and about my sensing, when I eat—those have been the key ingredients to the success. The fact that we have spoken on the basis of my entire life—especially my work life—and the fact that I have had the opportunity to express myself regarding the pressure I feel in relation to my work life has made a great difference. Along the way, between the sessions, I also gained insights from the past, which I had repressed, which have had a say in why I react as I do to demands, expectations, and perfectionism. This development has led to me being more attentive and not taking everything so seriously. Actually, you could say that I am now in a process, where I am practicing making mistakes and not complaining about it.

The coach has been inspiring me, listening to me, and challenging me—challenging me especially by questioning my understanding of how things are tied together—for example, that it is not a must to overeat because you feel that you are under pressure. The coach has also brought concrete suggestions to the table—for example, the good tip about trying the 5:2 diet, which turned out to fit well with my temperament, and is in line with the idea of not restricting yourself from the things you enjoy, but enjoying less of them.

An important quality of the coach has been setting firm requirements for what I could and couldn't do. For example, I wasn't allowed to drink cola. I suggested that it could be okay on weekends, but that was a no go. And it turned out that I quickly found joy in drinking sparkling water instead. Considering who I am as a person, it has been a plus to have pretty firm orders. Of course I could do as I pleased, but it was clear to me that if I was serious about the coaching I had to “obey orders.” When I first began with the coach, I drank 1½ L of cola every day. Today I drink it on special occasions, and weeks can go by where I don't drink it.

### The coach as a role model

It may sound as if it has been easy—and it both has and hasn't been. At times I have been surprised that it isn't harder, but there have also been times, where it has taken a lot of will power not to slip into “today it's okay to eat some sweets.” When having those thoughts, it has surprised me what role a coach plays. In these situations, the coach pops up in my head as a reminder. It is, of course, the feeling of having a responsibility toward the coach (and myself of course, but for me it is played out through the coach). The coach as a person has thus been a point of attention and been an inspiration in regard to keeping the deals we have made. The feeling that the coach has also given a part of himself—he can be under a similar pressure, but that doesn't mean he eats unhealthy things—has also been an inspiration.

### Timing

In regard to the relief I have achieved—“getting out of the dark hole”—I have afterwards thought about the timing of the process. Would I have achieved the same results if I had gone to see the coach sooner? Could I have avoided gaining the last five kilos? My answer is no, because the process has made it clear to me that it is also very much about being ready for it. Without my commitment and without me being ready to take action it won't succeed—even a motivating coach can't change that.

### The future

Naturally, I wish for it to pass quickly—meaning that I quickly will get even lighter and get rid of the last 10–12 kilos. But if I can do it the way I have just done it—lose it over the course of a year and a half, then that would definitely be preferred. Because it feels good that it is happening on the basis of me being aware of having a normal relationship with my food and what I consume.

## Conclusions

In this short section, I highlight a couple of conclusions that stand out from both my analysis of the sessions and Anna's evaluating reflections:

- *Health belief is a pure basis for change*: Perceived seriousness, perceived susceptibility to specific health and lifestyle issues, might lead to a growing awareness of health issues, but it did not help Anna understand herself and the complexity of her life situation as the basis for changing her lifestyle.
- *Being ready for change*: Anna stated that the time was ripe for her to change. Bohart and Talmann (2010) highlighted the client (in psychotherapy) as the central agent in the change process. With reference to different studies and authors, they see the client as the most important determinant of outcome. This can be assumed to also apply to coaching. Clients are not passive recipients in the dialogue; their willingness, their active involvement, and their way of making meaning of their life is pivotal for the progress and the effect of the intervention. In our last session, Anna emphasized that it is *hard work* to keep her ambitions alive and to continue to strive towards her goal.
- *Perceived support and encouragement from the social environment* and from the coach are fundamental for the client's willingness and ability to develop further and continue on the desired path. From this perspective, Anna's sister, as a supportive buddy, had a major influence on keeping Anna on the right track. They shared something important in their lives— Anna could phone her sister when things got hard (see also Weiner, 1998). The change in Anna's working environment (i.e., no overtime work), has been another key element which supported her weight loss ambitions.
- Anna mentioned the *commitment of the coach* (i.e., the determination to get her to drink mineral water instead of cola) as an important element for her development. This coach commitment was acknowledged by her as one of the central elements which kept her going. The collaborative perspective, also in regard to what worked well for her during the sessions (Miller, Duncan, Sorrell, Brown, & Chalk, 2006), was the key aspect of a fruitful and generative dialogue.

## Footnotes

1 The decision was made on the basis of an invitation by the guest editors of this issue, and after the first review of the suggestion of the topic and abstract provided by the author. The client of the study has given full consent to be the case of this study. On the basis of Danish law, an ethical approval for a social science study like this is not needed. Ethical committees simply do not treat social science research applications.

2 This term is used in systemic therapy and coaching (Cecchin, Lane, & Ray, 1993).
