# Corrigendum

**DOI:** 10.1080/17482631.2017.1400264

**Published:** 2017-11-12

Stelter. R. (2015). “I tried so many diets, now I want to do it differently”—A single case study on coaching for weight loss. International Journal of Qualitative Studies on Health and Well-being,10(1), 26925.

https://doi.org/10.3402/qhw.v10.26925

When the article was published online, the text ‘Health belief is a poor basis for change’ in the conclusion section was wrongly published as ‘Health belief is a pure basis for change’.

